# Sirt1 protects lupus nephritis by inhibiting the NLRP3 signaling pathway in human glomerular mesangial cells

**DOI:** 10.1515/biol-2022-1038

**Published:** 2025-04-25

**Authors:** Yu Zhao, Ai-Ping Zhang, Bei-Yan Bao, Heng Fan, Xu-Yan Yang

**Affiliations:** Department of Rheumatology, The Second Affiliated Hospital Zhejiang University School of Medicine, No. 88 Jiefang Road, Shangcheng District, Hangzhou, Zhejiang, P.R China; Department of Nephrology, Ningbo Yinzhou No. 2 Hospital, Ningbo, Zhejiang, P.R China; Department of Intensive Care Unit, The First Affiliated Hospital of Ningbo University, No. 59 Liuting Road, Ningbo, Zhejiang, P.R China

**Keywords:** Sirt1, NLRP3, lupus nephritis, human glomerular mesangial cells, signaling pathway

## Abstract

Lupus nephritis (LN) is the most common and lethal complication of systemic lupus erythematosus. We aimed to explore the protective effect of Sirtuin1 (Sirt1) on LN by regulating the NLRP3 signaling pathway in human glomerular mesangial cells (GMCs). We collected clinical samples from patients with LN, detected Sirt1 protein and mRNA expression using biochemical methods, cultured GMCs *in vitro*, evaluated levels of oxidative stress, cell apoptosis, and mitochondrial damage, and analyzed the expression of NLRP3 pathway proteins. Our results demonstrated that Sirt1 protein and mRNA were downregulated in the renal tissue of LN patients, and LN serum induced an increase in oxidative stress, cell apoptosis, and mitochondrial damage in GMCs while activating the NLRP3 signaling pathway. Upregulation of Sirt1 inhibited LN serum-induced oxidative stress in GMCs, reduced the number of cell apoptosis, and stabilized mitochondrial structure and function. Moreover, Sirt1 overexpression inhibited the expression of NLRP3 pathway proteins. Our findings suggest that Sirt1 may protect LN by inhibiting the NLRP3 signaling pathway in GMCs.

## Graphical abstract

**Figure:**
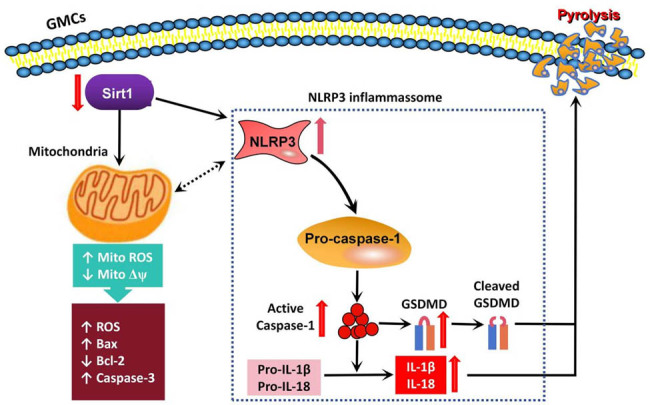

## 1 Introduction

Systemic lupus erythematosus (SLE) is a chronic autoimmune disease that involves multiple systems, and the kidney is the most common target organ [1]. According to statistics, 40–60% of SLE patients have lupus nephritis (LN), which is a lethal complication [2]. The etiology of LN is related to genetic, immune, endocrine, and infectious factors [3]. The pathological essence of LN is immune complex glomerulonephritis, including abnormal deposition of immune complexes, activation of inflammatory reactions leading to mesangial proliferation, extracellular matrix deposition, interstitial fibrosis, etc. [4,5]. However, the mechanisms underlying the regulation of inflammatory and immune responses during LN have not been fully elucidated, resulting in suboptimal clinical treatment outcomes.

Sirtuin1 (Sirt1) is a deacetylase that relies on nicotinamide adenine dinucleotide (NAD^+^), and which can deacetylate various transcription factors and participate in glucose and lipid metabolism, organ metabolism, and oxidative stress, playing a protective role in various diseases [6]. Sirt1 can deacetylate specific lysine residues in histones, related transcription factors, and signaling molecules, participating in the regulation of various metabolic pathways, including cell proliferation and differentiation, glucose homeostasis, fat metabolism, cell apoptosis, aging, and longevity [7]. The decrease in Sirt1 activity leads to high activation of T lymphocytes, enhanced immune response, and the inability of T lymphocytes to tolerate their own antigens, resulting in autoimmune phenomena such as anti-nuclear antibodies, systemic lymphocyte infiltration, and immunoglobulin deposition [8]. A study has found a correlation between Sirt1 levels in the urine of SLE patients and the severity of LN, suggesting that Sirt1 may become a new target of LN [9].

LN is considered a prototype autoimmune disease characterized by abnormal reactions of T and B cells, and excessive antibody production and immune complex formation are the main pathogenesis [10]. Lymphocyte dysfunction, accumulation of autoantibodies, impaired clearance of circulating immune complexes, and apoptotic cells are characteristics of LN [11]. However, the role of Sirt1 in the pathogenesis of LN is still unclear. Therefore, this study aimed to investigate the role of Sirt1 in the pathophysiological mechanisms of LN, providing new references for clinical patients.

## 2 Materials and methods

### 2.1 Patients and samples

We selected 10 LN patients, regardless of gender, who were admitted to Ningbo Yinzhou No. 2 Hospital from January 2023 to December 2023. The inclusion criteria: (1) meeting the diagnosis of SLE; (2) diagnosed as LN through renal puncture biopsy; and (3) complete clinical and pathological data. Ten patients who underwent nephrectomy due to trauma during the same period were included in the normal control (NC) group. The data collection included: serum creatinine (Scr), blood urea nitrogen (BUN), plasma renal injury molecule-1 (pKIM-1), 24 h urine protein quantification, and pathological characteristics. Clinical sample collection and preservation: (1) blood sample: 3 mL of fasting elbow vein blood, allowed to settle for 0.5 h, naturally coagulated, then centrifuged in a centrifuge at a speed of 3,000 rpm for 10 min, serum was separated and stored in a −80°C freezer; (2) kidney tissue: kidney tissue from LN patients undergoing renal puncture and normal kidney tissue from SLE patients undergoing surgical resection were divided into two parts. One part was frozen in liquid nitrogen for 0.5 h and stored in a −80°C refrigerator, while the other part was fixed with paraformaldehyde and embedded in paraffin for preservation.


**Informed consent:** Informed consent has been obtained from all individuals included in this study.
**Ethical approval:** The research related to human use has been complied with all the relevant national regulations and institutional policies and in accordance with the tenets of the Helsinki Declaration and has been approved by the Ethics Committee of the Ningbo Yinzhou No. 2 Hospital (Approval No. Yin2-2023-051).

### 2.2 Cell culture

We cultured the human glomerular mesangial cells (GMCs) (JI Ning Bio Co., Beijing, China) in a 37°C, 5% CO_2_ incubator and observed the morphological changes in the cells every day. After 72 h, we collected the cells for subsequent experiments. We divided GMCs into the control, LN, LN + siSirt1, and LN + oeSirt1 groups. (1) The control group: no processing was done; (2) the LN group: we added serum drops from LN patients to cell culture medium (5%) for co-cultivation [12]; (3) co-culture Sirt1 silenced GMCs cells with cell culture medium containing LN patient serum (5%); and (4) co-culture GMCs cells overexpressing Sirt1 with cell culture medium containing LN patient serum (5%). Subsequent experiments began on the 7th day of cell co-culture.

### 2.3 Pathological injury

We followed the routine procedure of fixing renal tissue with 4% formaldehyde and embedding it in paraffin sections. Then, hematoxylin-eosin staining was performed, and pathological changes in renal tissue were observed and photographed under an optical microscope.

### 2.4 Transfection of siRNA and lentiviral plasmids

We operated according to the product reagent kit and instructions. The titer of lentivirus was 5 × 10^8^ TU/mL. After 72 h of transfection, detection showed that good transfection and expression efficiency had been achieved when the multiple of infection value was 10.

### 2.5 Immunohistochemistry

The kidney tissue embedded in paraffin from a patient was taken and subjected to an immunohistochemical staining kit (Boster Co., Wuhan, China) for the experiment. Incubated Sirt1 primary antibody at 4°C overnight, DAB staining, and hematoxylin staining. Observing under a microscope, took five random fields of view under a high-power microscope to calculate the average optical density value. The cytoplasm was stained brown-yellow as positive cells, and the percentage of positive cells in the field of vision was calculated.

### 2.6 Inflammation level

We followed the instructions and used the Elisa kit (Xitang Co., Shanghai, China) to detect the inflammatory cytokine IL-1 and IL-18 expression in GMCs.

### 2.7 Oxidative stress

We used a DCFH-DA probe to determine intracellular reactive oxygen species (ROS). We collected single-cell samples and detected the fluorescence value. We measured the levels of oxidase in GMCs according to the instructions, including malondialdehyde (MDA) (Cytogen Co., Suzhou, China), glutathione peroxide (GSH-Px) (Cytogen Co., Suzhou, China), and superoxide dismutase (SOD) (Cytogen Co., Suzhou, China) levels, and plotted the standard curve.

### 2.8 Mitochondrial morphology and function

We inoculated logarithmic growth stage cells into a culture bottle, prepared routine transmission electron microscopy samples, and observed the subcellular structure of GMCs under transmission electron microscopy. We followed the instructions of the reagent kit to detect the levels of mitochondrial complex enzymes in GMCs and plotted a standard curve.

### 2.9 Mitochondrial membrane potential

We used the mitochondrial membrane potential detection kit (JC-1) to detect Δψm. Cells were collected, resuspended, and mixed with JC-1 staining solution. Incubated at 37°C in a cell culture box for 20 min, washed with JC-1, added cell culture solution, and observed under a fluorescence microscope.

### 2.10 Flow cytometry

Suspended GMCs were taken, centrifuged at 800 rpm for 5 min, discarded the supernatant, added 70% ethanol, overnight at 4°C, centrifuged at 1,000 rpm for 5 min, discarded the supernatant, added 5 µL RNaseA, digested at 37°C for 1 h, added 50 mg/mL propidium iodide, and stained in dark at 4°C for 1 h, analyzed on EPICS XL flow cytometry.

### 2.11 Real time quantitative polymerase chain reaction

We used the TRIzol extraction kit (Sigma Co., Shanghai, China) to extract total RNA from renal tissue and GMCs for reverse transcription reaction. Reverse transcription reaction conditions were as follows: 37°C for 15 min, 85°C for 5 s, and stored at 40°C. Polymerase chain reaction (PCR) conditions were as follows: 95°C for 30 min, 1 cycle; 95°C for 5 s, 60°C for 30 s, a total of 40 cycles; 95°C for 15 s, 60°C for 1 min, 95°C for 15 s, 1 cycle. We applied the Roche 480 II PCR instrument to react with the reverse transcripts of each group and obtained Mean CT values, using the 2^−ΔΔCt^ method for data analysis.

### 2.12 Western blotting

We extracted cytoplasmic proteins and nuclear proteins and used bovine serum albumin as the standard. We used the bicinchoninic acid method to perform the protein quantification. We took an appropriate amount of protein sample, performed 10% SDS-PAGE electrophoresis (300 V, 40 min), transferred the membrane at 300 mA for 2 h, and placed it in 5% skim milk for shaking and sealing, primary antibody (Boster Co., Wuhan, China) overnight at 4°C. After the membrane was repeatedly washed with TBST, it was incubated with the secondary antibody. The membrane was placed on a shaker and shaken at room temperature for 1 hour. After further washing the membrane with TBST, enhanced chemiluminescence was used for color development. Finally, the absorbance values of individual bands were measured using a gel image analysis system to conduct quantitative analysis.

### 2.13 Statistical analysis

All statistical analyses were conducted using GraphPad Prism 8.25 software. The data were expressed as mean ± standard deviation, one-way analysis of variance was used for inter-group comparisons, and the Tukey method was used for post hoc test analysis for intra-group pairwise comparisons. When *P* < 0.05, the difference was considered statistically significant.

## 3 Results

### 3.1 Low expression of Sirt1 in renal tissue of LN patients

**Figure 1:**
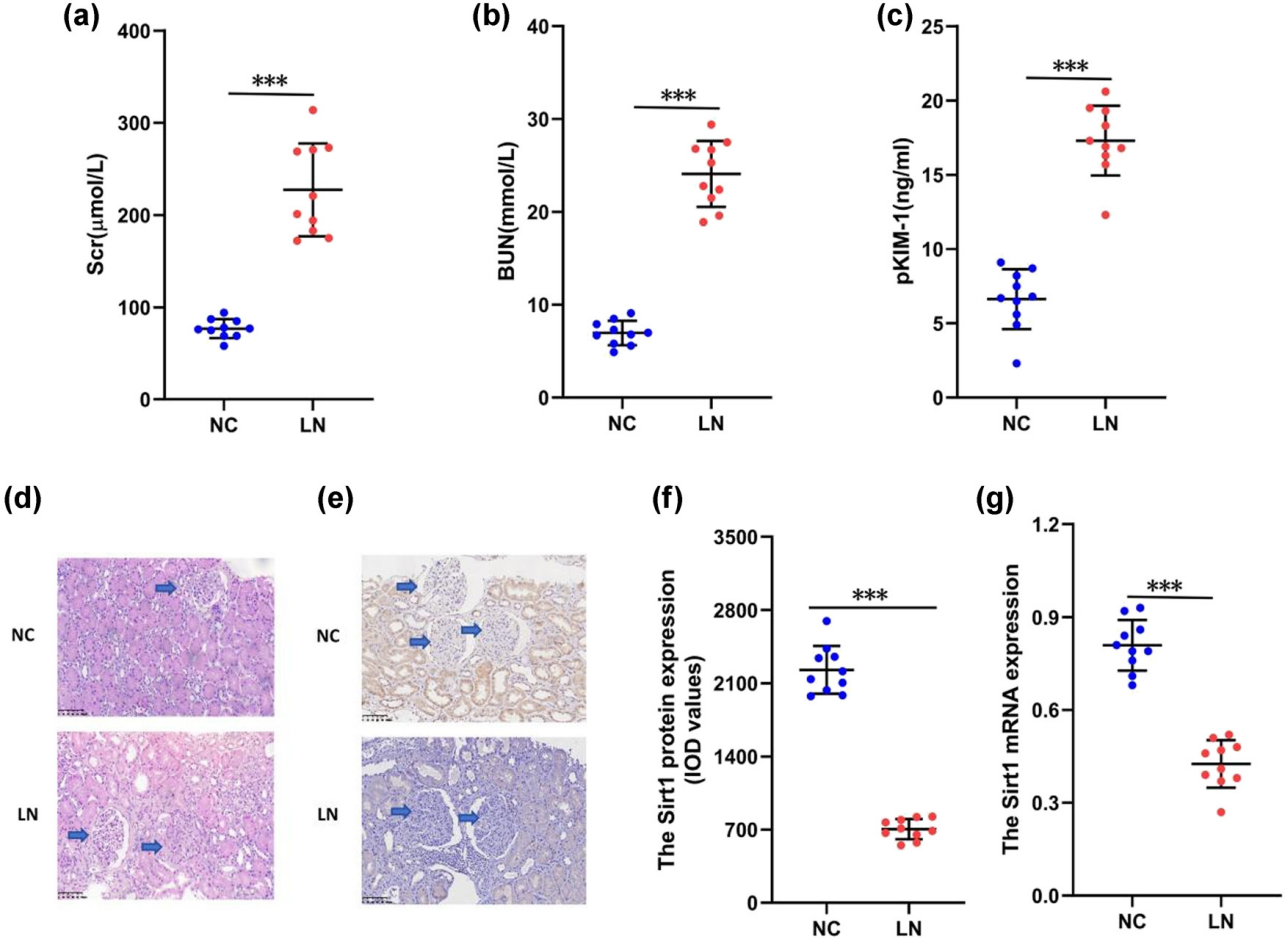
The expression of Sirt1 in renal tissue of LN patients. (a) the level of Scr; (b) the level of BUN; (c) the level of pKIM-1; (d) renal pathology (×400); (e) the expression of Sirt1 protein (×400); (f) the level of Sirt1 protein; and (g) the expression of Sirt1 mRNA. Compare with the LN, *P* < 0.05, *P* < 0.01, ***P < 0.001. Sirt1, sirtuin1; Scr, serum creatinine; BUN, blood urea nitrogen; pKIM-1, plasma renal injury molecule-1.

We included 10 LN patients and 10 NC patients. There were no significant differences in baseline characteristics between the two groups, such as age, gender, weight, and the proportion of hypertension and diabetes. The renal function indicators, including Scr, BUN, and pKIM-1 levels, were significantly increased in all LN patients, with segmental sclerosis, interstitial fibrosis, and inflammatory cell infiltration in the renal tissue (Figure 1a–d). Compared with NC patients, the expression of Sirt1 protein and mRNA in the renal tissue of LN patients was significantly reduced (Figure 1e–g). We had preliminarily determined that Sirt1 was low expressed in the renal tissue of LN patients. The abnormal expression of Sirt1 in GMCs of LN patients may be an important cause of the disease, so we focus on exploring the mechanism of Sirt1 in GMCs.

### 3.2 Sirt1 inhibits oxidative stress in GMCs

**Figure 2:**
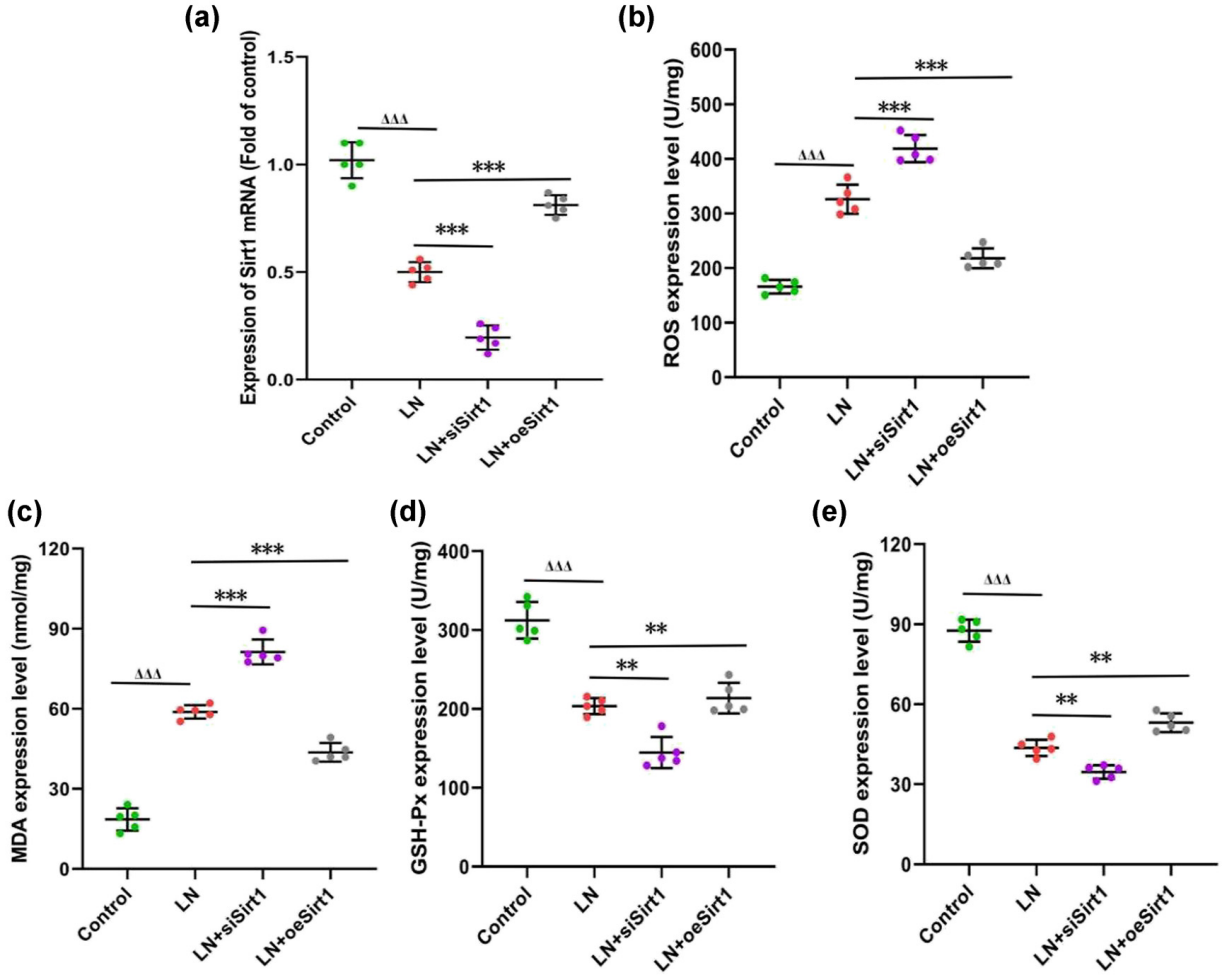
Sirt1 inhibits oxidative stress in GMCs. (a) expression of Sirt1 in different groups; (b) the level of ROS in GMCs; (c) the level of MDA; (d) the level of GSH-Px; and (e) the level of SOD. Compare with the Control, ^ΔΔΔ^ *P* < 0.001; compare with the LN, **P < 0.01, ***P < 0.001. GMCs, human glomerular mesangial cells; ROS, reactive oxygen species; MDA, malondialdehyde; GSH-Px, glutathione peroxide; SOD, superoxide dismutase.

Oxidative stress is one of the main pathological changes of LN and the fundamental cause of rapid deterioration of the disease [13]. On the 7th day of culturing GMCs in LN serum, we found that intracellular ROS levels were significantly increased, and Sirt1 silencing led to a more pronounced increase (Figure 2a and b). However, overexpression of Sirt1 reversed the sharp increase in ROS levels caused by LN (Figure 2a and b). There were similar results in monitoring oxidase levels. In the LN group, the oxidase MDA increased, while the antioxidant enzymes including GSH-Px and SOD decreased (Figure 2c–e). Although Sirt1 silencing promoted more significant abnormal expression of oxidase and antioxidant enzymes, overexpression of Sir1 reversed this phenomenon (Figure 2c–e). It could be seen that the antioxidant stress effect of Sirt1 in LN was crucial for its protection of GMCs.

### 3.3 Sirt1 inhibits apoptosis of GMCs

**Figure 3:**
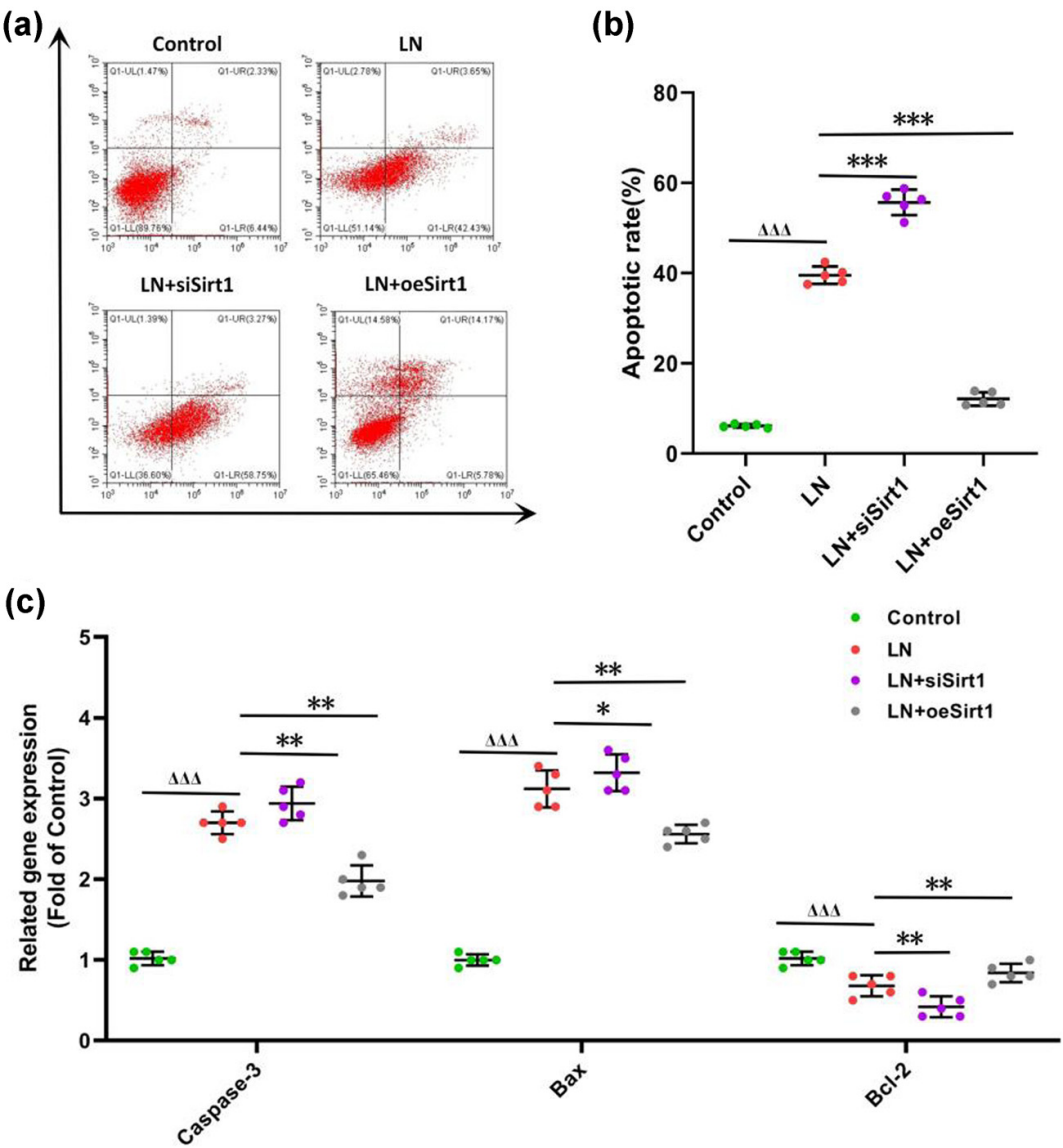
Sirt1 inhibits apoptosis of GMCs. (a) apoptosis of GMCs; (b) apoptotic rate of GMCs; and (c) the expression of apoptosis-related genes. Compare with the Control, ^ΔΔΔ^ *P* < 0.001; compare with the LN, *P < 0.05, **P < 0.01, ***P < 0.001. GMCs, human glomerular mesangial cells.

The increase in cell apoptosis can lead to a rapid deterioration of LN, resulting in an irreversible decline in renal function [14]. We found that the number of apoptotic cells in GMCs cultured in the serum of LN patients increased, and Sirt1 silencing increased the number of apoptotic cells. However, overexpression of Sirt1 resulted in a decrease in the number of cell apoptosis (Figure 3a and b). In measuring the expression of apoptosis-related genes in GMCs, we found that in GMCs cultured with serum from LN patients, pro-apoptotic genes including Bax and Caspase-3 were increased, while the apoptosis inhibitory gene Bcl-2 was reduced (Figure 3c). Silencing Sirt1 leads to more significant changes in apoptosis-related genes, while overexpression of Sirt1 could stabilize this phenomenon (Figure 3c). Therefore, we considered that Sirt1 might protect LN by inhibiting GMCs apoptosis.

### 3.4 Sirt1 protects the mitochondrial function of GMCs

**Figure 4:**
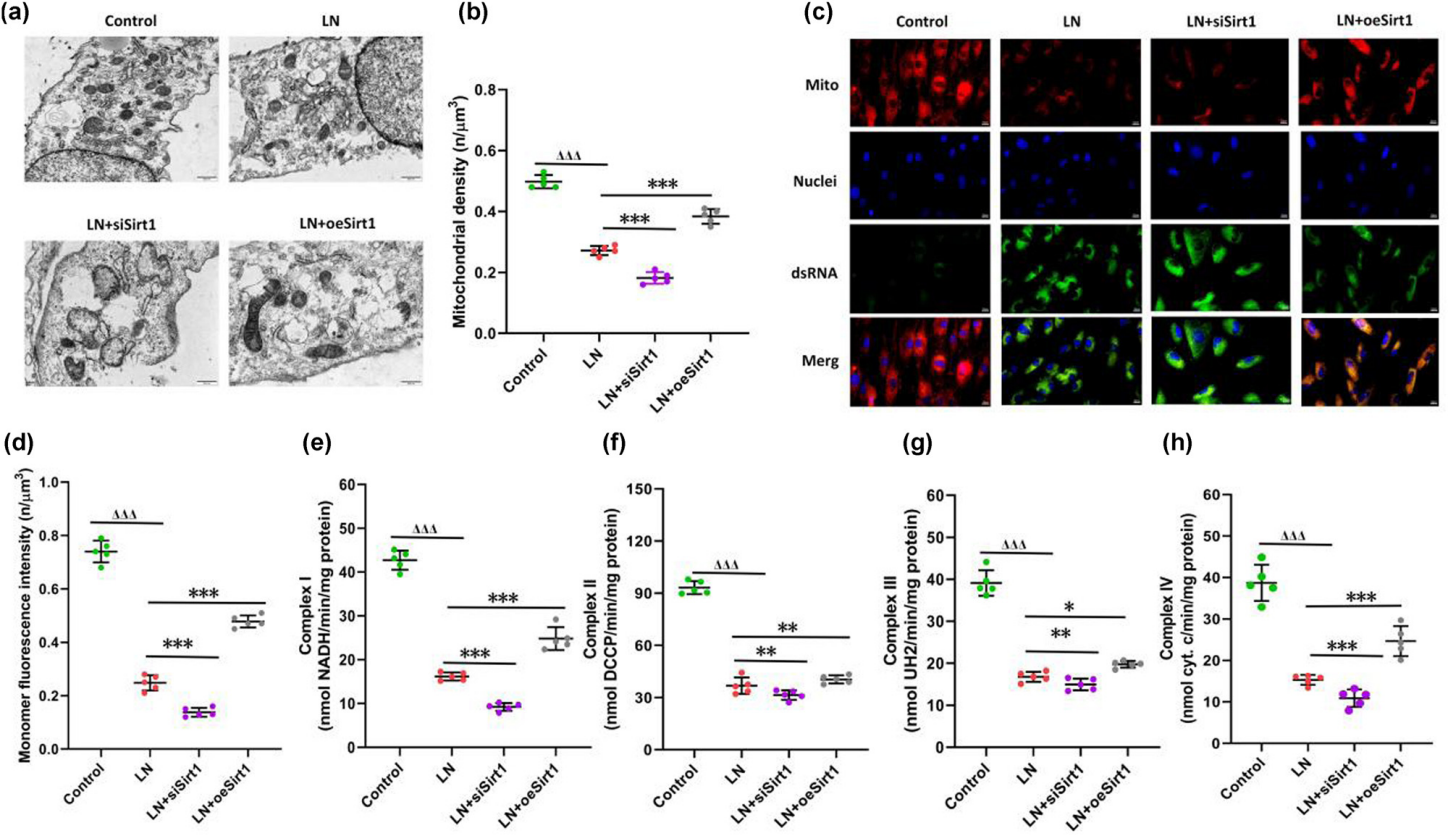
Sirt1 protects the mitochondrial function of GMCs. (a) observation of mitochondrial morphology; (b) changes in mitochondrial density; (c) changes in mitochondrial membrane potential; (d) quantitative changes in mitochondrial membrane potential; the changes in mitochondrial complex enzymes I (e), II (f), III (g), and IV (h). Compare with the control, ^ΔΔΔ^ *P* < 0.001; compare with the LN, *P < 0.05, **P < 0.01, ***P < 0.001. GMCs, human glomerular mesangial cells.

Mitochondria are the site of oxidative processes and key organelles involved in oxidative stress damage [15]. The decrease in mitochondrial membrane potential is a landmark event in the early stage of cell apoptosis, and the collapse of mitochondrial membrane potential will make it difficult to reverse cell apoptosis [16]. In LN serum-induced GMCs, the overall morphology of mitochondria was irregular, with membrane ruptured and decreased membrane potential (Figure 4a–d). Sirt1 silencing induced overall mitochondrial morphology disorder, with most of the mitochondrial membrane destroyed and membrane potential reduced (Figure 4a–d). However, overexpression of Sirt1 inhibited LN-induced mitochondrial damage in GMCs, protected the stability of the intracellular mitochondrial membrane, and increased membrane potential (Figure 4a–d). Moreover, in the exploration of mitochondrial complex enzymes in GMCs, we found that overexpression of Sirt1 partially restored the activity of mitochondrial complex enzymes, with the most significant effects on mitochondrial complex enzymes I and IV (Figure 4e–h). We believed that Sirt1 effectively protected LN by stabilizing mitochondrial structure and function.

### 3.5 Sirt1 inhibits the NLRP3 pathway in GMCs

**Figure 5:**
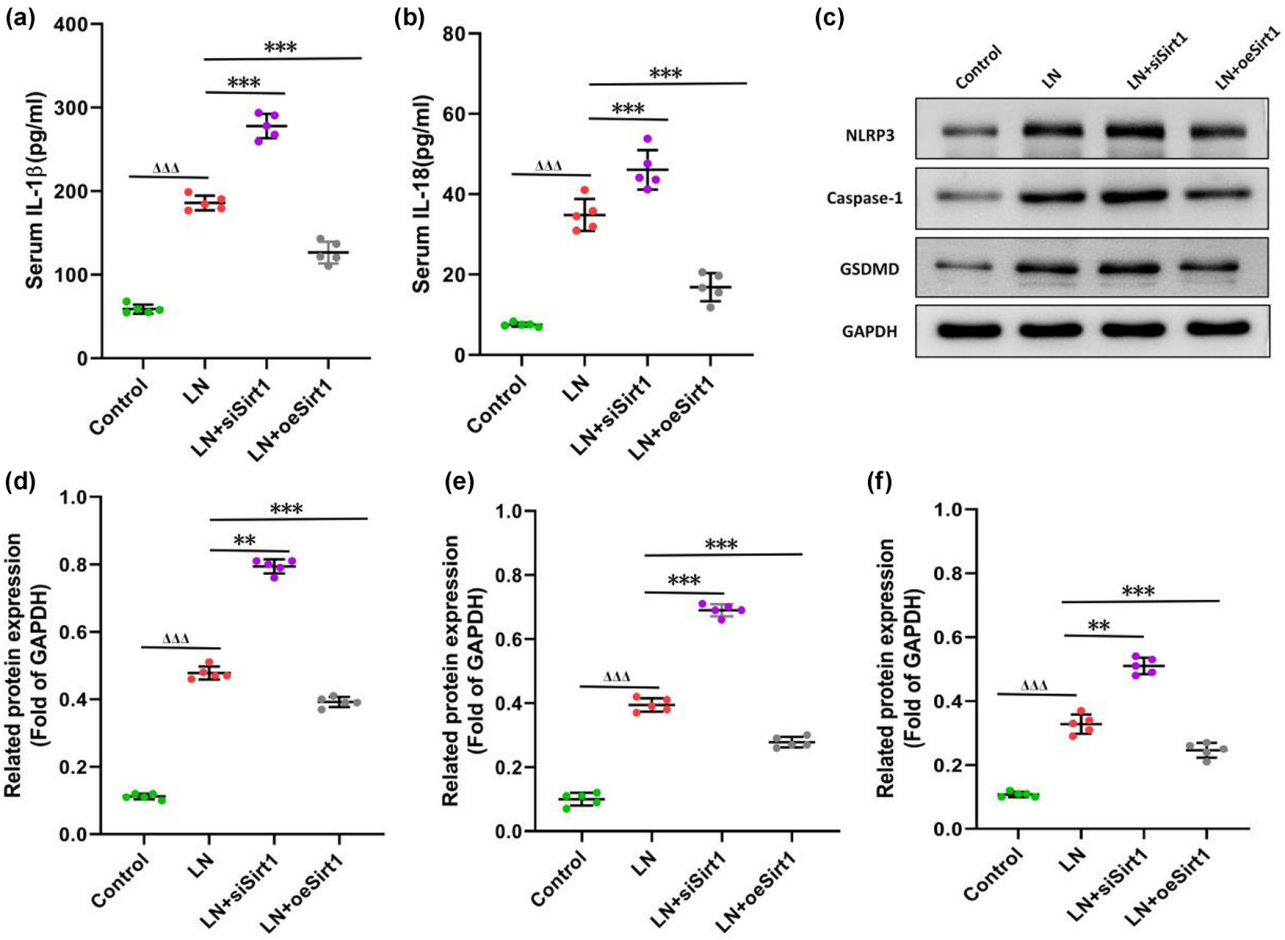
Sirt1 inhibits the NLRP3 pathway in GMCs. (a) the level of IL-1β; (b) the level of IL-18; (c) the expression of NLRP3 pathway proteins; (d) the protein levels of NLRP3; (e) the protein levels of Caspase-1; and (f) the protein levels of GSDMD. Compare with the control, ^ΔΔΔ^ *P* < 0.001; compare with the LN, **P < 0.01, ***P < 0.001. GMCs, human glomerular mesangial cells.

The NLRP3 pathway is a key inflammatory regulatory pathway and an important pathway for the evolution of LN [17]. We found that LN serum induced the inflammatory cytokine IL-1β and IL-18 increased in GMCs, while NLRP3 pathway proteins including NLRP3, Caspase-1, and GSDMD were also elevated (Figure 5a–f). Silence of Sirt1 leads to a more significant increase in IL-1 β, IL-18, and NLRP3 pathway proteins (Figure 5a–f). However, overexpression of Sirt1 leads to a decrease in IL-1 β and IL-18, and all NLRP3 pathway proteins were also significantly reduced (Figure 5a–f). Our results indicated that Sirt1 might protect LN by inhibiting the NLRP3 inflammatory pathway.

## 4 Discussion

SLE is a systemic disease caused by autoimmune dysfunction, with LN being the most common and severe organ damage in SLE. As a type of chronic kidney disease, LN has the characteristics of alternating disease activity and remission, long disease course, and multiple complications. Sirt1 belongs to the protein deacetylase family and is involved in regulating glucose and lipid metabolism, inflammatory response, cell aging and apoptosis, oxidative stress, and tumor formation [18]. Sirt1 regulates cell activity through transcriptional factor deacetylation, thereby exerting biological functions. In the present study, our results showed that the expression of Sirt1 protein and mRNA in the renal tissue of LN patients was significantly lower than that of NC patients, and Sirt1 protein expression was mainly concentrated in renal tubules and GMCs.

A study showed that oxidative stress plays an important role in the pathogenesis of LN [19]. ROS is produced by cells during normal processes, and the antioxidant system of cells minimizes the damage. Brezovec et al. [20] found that Sirt1 is also involved in the regulation of ROS *in vivo*, and regulating Sirt1 may become a strategy to combat the generation of ROS. We found that overexpression of Sirt1 could inhibit the generation and release of ROS in GMCs, inhibit oxidase activity, and promote antioxidant enzyme activity, demonstrating its potential role in stabilizing oxidative stress responses in GMCs. Our study further confirms the above conclusion. Moreover, Sirt1 affects the upstream pathway of PGC-1ɑ to alleviate high glucose-induced mitochondrial dysfunction in renal tubular cells and reduce oxidative stress damage and cell apoptosis [21].

In the present study, we indicated that activating Sirt1 could inhibit LN-induced apoptosis of GMCs cells, downregulate the expression of apoptosis-related genes, and upregulate the level of anti-apoptotic genes, playing a key role in the mechanism of GMCs cell apoptosis during LN. Pan et al. [22] found that Sirt1 perceives changes in the intracellular environment through the redox reaction of NAD^+^/NADH and subsequently deacetylates FOXO1 and PGC-1ɑ, thus enhancing cell vitality and participating in anti-apoptotic, anti-inflammatory, and antioxidant stress processes. Jalgaonkar et al. [23] showed that curcumin can regulate the Sirt1/FOXO1 pathway by inhibiting the acetylation of FOXO1, alleviate oxidative damage caused by type 2 diabetes, and protect cardiomyocytes from apoptosis. In addition, Maorui isoflavone glycoside acts on neuronal cells after cerebral ischemia-reperfusion, upregulating Sirt1, FOXO1, PGC-1ɑ, and Bcl-2, while downregulating Bax, indicating that the flavonoid glycoside alleviates neuronal apoptosis and oxidative stress through the Sirt1/FOXO1/PGC-1ɑ signal pathway [24]. Resveratrol as an agonist of Sirt1 can be activated by activating the Sirt1/PGC-1ɑ pathway reducing apoptosis of renal tubular cells caused by high glucose [25].

Mitochondria are important organelles for maintaining cells and the main site for the formation of adenosine triphosphate. During the induction phase of cell apoptosis, characteristic changes in the structure and function of mitochondria begin to occur, including the loss of tightly arranged and orderly folded mitochondrial cristae, as well as the filling of concentrated matrix in membrane gaps, while the size changes of mitochondria themselves are not significant [26]. However, the specific pathway by which the increase in mitochondrial matrix density induces cell apoptosis is still unclear. In the present study, we suggested that upregulation of Sirt1 could maintain the stability of mitochondrial structure and function in GMCs, thereby protecting the integrity of the mitochondrial respiratory chain and reducing the occurrence of cell apoptosis. The apoptosis mechanism induced by mitochondrial damage mediated by Sirt1 also participates in the pathological process of diabetes nephropathy and can improve symptoms or delay disease progression by regulating its pathway [27].

NLRP3, as an intracellular pattern recognition receptor, plays an important role in innate immunity [28]. After NLRP3 inflammasome is activated, it can self-activate Caspase-1, ultimately leading to the maturation and secretion of IL-1β and IL-18, thereby amplifying the inflammatory response [29]. Among the substrates regulated by Sirt1, key transcription factors associated with renal disease progression include NF-κB, STAT3, and p53, among which NF-κB is a transcription factor closely related to inflammation regulated by Sirt1 [30,31,32]. In this study, we found that overexpression of Sirt1 led to a decrease in IL-1β and IL-18, and all NLRP3 pathway proteins were also significantly reduced. We inferred that Sirt1 might protect LN by inhibiting the NLRP3 signaling pathway.

Our study has the following limitations. First, we found that Sirt1 was expressed on both GMCs and renal tubular epithelial cells, but we only studied GMCs. The relevant mechanisms still need further clarification. Second, we found that the expression of Sirt1 was low in LN serum-induced GMCs, and further research would be needed to investigate the related pathways mediated by Sirt1 in conditionally knockout mice. Third, further research will be needed to confirm whether Sirt1 inhibitor can affect the NLRP3 signaling pathway and improve LN; this will be the focus of future studies.

## 5 Conclusion

Sirt1, as a key gene regulating T-lymphocyte immune tolerance, protects LN by reducing oxidative stress response, inhibiting cell apoptosis, and stabilizing mitochondrial morphology and function. The specific mechanism may be related to its regulation of the NLRP3 signaling pathway.
